# Modelling the spatial distribution of five natural hazards in the context of the WHO/EMRO Atlas of Disaster Risk as a step towards the reduction of the health impact related to disasters

**DOI:** 10.1186/1476-072X-6-8

**Published:** 2007-03-07

**Authors:** Zine El Abidine El Morjani, Steeve Ebener, John Boos, Eman Abdel Ghaffar, Altaf Musani

**Affiliations:** 1 Emergency Preparedness & Humanitarian Action, World Health Organization Regional Office for the Eastern Mediterranean, P.O. Box 7608, Nasr City (11371), Cairo, Egypt; 2 Evidence and Information for Policy, World Health Organization, 20 av. Appia, 1211 Geneva 27, Switzerland

## Abstract

**Background:**

Reducing the potential for large scale loss of life, large numbers of casualties, and widespread displacement of populations that can result from natural disasters is a difficult challenge for the individuals, communities and governments that need to respond to such events.

While it is extremely difficult, if not impossible, to predict the occurrence of most natural hazards; it is possible to take action before emergency events happen to plan for their occurrence when possible and to mitigate their potential effects.

In this context, an Atlas of Disaster Risk is under development for the 21 Member States that constitute the World Health Organization's (WHO) Eastern Mediterranean (EM) Region and the West Bank and Gaza Strip territory.

**Methods and Results:**

This paper describes the Geographic Information System (GIS) based methods that have been used in order to create the first volume of the Atlas which looks at the spatial distribution of 5 natural hazards (flood, landslide, wind speed, heat and seismic hazard).

It also presents the results obtained through the application of these methods on a set of countries part of the EM Region before illustrating how this type of information can be aggregated for decision making.

**Discussion and Conclusion:**

The methods presented in this paper aim at providing a new set of tools for GIS practitioners to refine their analytical capabilities when examining natural hazards, and at the same time allowing users to create more specific and meaningful local analyses.

The maps resulting from the application of these methods provides decision makers with information to strengthen their disaster management capacity. It also represents the basis for the reflection that needs to take place regarding populations' vulnerability towards natural hazards from a health perspective.

## Background

The impact of natural disasters over the last decade has resulted in many lives lost and livelihoods destroyed. Recent disasters in the EM Region such as the earthquakes in Pakistan (2005) and Islamic Republic of Iran (2003), the drought in the Horn of Africa (2006), and landslides in Yemen (2005), have tested the capacities of Member States as well as national and international humanitarian agencies to provide quick and effective assistance. These disasters have resulted in significant mortality, morbidity and disability among the affected populations. The 2005 earthquake in Pakistan in particular resulted in more than 70,000 dead, 73,000 injured and more than 3 million survivors left at risk for additional mortality and morbidity. In addition to the direct impact on the health of affected populations, these and other disasters resulted in some situations to further increase vulnerability post an event by the destruction of homes, businesses and vital infrastructure like hospitals. Access and availability of basic health services, shelter, food, clean water and sanitation which may have been destroyed or reduced as a resulted of a major disaster are critically needed to avert excess morbidity and mortality among survivors.

Many studies have already demonstrated how poverty in many communities only increasing as a result of disasters. Furthermore, from a health perspective those individuals (or those communities which represent the high burden of communicable and non-communicable diseases) who may be chronically ill having diabetes, cardiovascular disease, or HIV/AIDs or pregnant women who may have complications will need to have access to life saving services of which may not exist as a result of an emergency. The post traumatic stress and mental illness following a large scale disaster has also addressed a key public health priority in the event of an emergency.

Subsequently, many of the affected countries and communities in the Eastern Mediterranean Region are now calling for better disaster preparedness and mitigation programs to avert the adverse effects of major disasters. This has been clearly echoed by many Member States during recent World Health Assemblies and meetings of the Regional Committee for the Eastern Mediterranean where several resolutions (WHA 58.1, WHA 59.22, and EM/RC49/R.7) have tasked WHO with improving national and local capacities.

In addition, the ability to determine disaster risk for a region and its resident populations will strengthen its disaster management capacity by providing the information necessary to decision makers to: advocate for resources to improve emergency preparedness and mitigation; supporting emergency response; help to identify, plan and prioritize areas for mitigation activities to minimize the effects of natural hazards; and provide a springboard for additional disaster management and recovery activities.

Nevertheless, natural disaster risk assessment is a complex task, involving a wide variety of processes which require large amounts of spatial and temporal thematic data and information coming from disparate sources. In this context, geography and Geographic Information Systems (GIS) can provide an ideal platform for the integration of the different data, their analysis and, ultimately, the development of disaster risk models for a region and its resident populations.

Globally, previous work on the distribution of natural hazards using GIS has been developed and illustrated in the Natural Disaster Hotspots (NDH) project implemented by Columbia University and the World Bank under the umbrella of the ProVention Consortium [1]. Additionally, the UN Development Programme (UNDP), in partnership with UNEP-GRID, examined the quantification of disaster risk through the development of a Disaster Risk Index (DRI) for the International Secretariat on Disaster Reduction (ISDR) Inter-Agency Task force on Disaster Reduction [2,3]. At a more local level, a number of studies have been undertaken within the EM Region mainly in connection with landslide and floods [4-7].

While the data produced through the NDH project represents a starting point for assessing natural disaster risk in the EM Region and the West Bank and Gaza Strip territory, some limitations have been identified regarding their potential use for prioritizing mitigation and preparedness activities. These limitations include the fact that the resolution at which the maps have been produced might not be detailed enough for effective decision making and that some important factors have not been taken into account when modelling the distribution of the hazards.

Hence, with the objective of reducing risks to vulnerable populations, the World Health Organization (WHO) Eastern Mediterranean Regional Office (EMRO) decided to look at overcoming some of these limitations by complementing the work already done with a research-oriented project.

**Figure 1:**
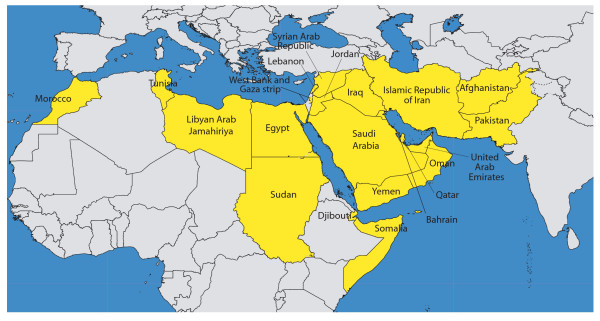
The WHO EM Region and the West Bank and Gaza Strip territory.

The outcome of this work will be an Atlas concentrating on five natural hazards (floods, heat, seismic hazard, wind speed and landslides) at a resolution of one square kilometer for the 21 countries that are part of this region and the West Bank and Gaza Strip territory (Figure 1).

WHO developed the first volume of the Atlas in order to encourage and stimulate Ministries of Health and other stakeholders within the health community to improve their disaster management capacity. More specifically it is expected that the Atlas will be used as a tool to: advocate for resources to improve disaster preparedness in the health sector; aid emergency response measures through better baseline information; assist in identifying, planning and prioritizing areas for mitigation activities to minimize the effects of natural hazards and provide a springboard for transitional and early recovery activities after an emergency.

In addition to that, risk identification and related activities being one of the five priorities for action of the Hyogo Framework for Action (HFA) [8] this Atlas will also contribute to the implementation of the strategy for disaster reduction.

Using GIS, three components will first be mapped for each of the EM countries and the West Bank and Gaza Strip territory: the distribution of potential hazards, the distribution of population (the element at risk) and the distribution of the population's vulnerability. These components will be combined according to the following formula, developed by UNDRO [9], in order to obtain a measure of the risk:

Risk = Hazard × Element at Risk × Vulnerability

In this context, the United Nations International Strategy for Disaster Reduction (UNISDR) [10] defines a hazard as "a potentially damaging physical event, phenomenon or human activity that may cause the loss of life or injury, property damage, social and economic disruption or environmental degradation" each hazard being characterized by its location, intensity, frequency and probability.

It then defines vulnerability as "the conditions determined by physical, social, economic, and environmental factors or processes, which increase the susceptibility of a community to the impact of hazards."

And finally, risk as being "the probability of harmful consequences, or expected losses (deaths, injuries, property, livelihoods, economic activity disrupted or environment damaged) resulting from interactions between natural or human-induced hazards and vulnerable conditions". As presented in the above formula we also include here the concept of exposure to refer particularly to the physical aspects of vulnerability (population in this case).

Each of these components (hazard, vulnerability and risk) will be the topic of a separate volume of the Atlas. The objective of the present paper is to address the data and models used or developed in order to obtain the spatial distribution of the selected natural hazards. The regional and country level maps for all the 21 countries and the West Bank and Gaza Strip territory that resulted from this work forms the first volume of this Atlas, which was launched during the 17^th ^United Nations Regional Cartographic Conference for Asia and the Pacific (UNRCC-AP) [11] and which will be published in spring 2007. This Atlas can be requested from the WHO/Eastern Mediterranean Regional Office by contacting the last co-author of this paper.

## Data

The application of the models presented in this paper has been performed on geographic layers compiled and homogenized in order to cover the 21 countries part of the EM Region and the West Bank and Gaza Strip territory and to present specification as close as possible to the ones listed here:

• 1:1,000,000 scale for the vector layers

• 1 kilometer resolution for the raster layers

• Arcview shapefile or grid format

• Coverage matching the international boundary data set prepared by the UN

In order to give the emergency management community the opportunity to apply the models to other regions and taking into account that some countries may have incomplete data at disposal, priority was given to using freely accessible global data sets corresponding to the specifications mentioned above. When this was not possible, other sources of information were used. It is important to mention that the methods developed and used in this project allow for the replacement of any of the data sources with datasets that are of higher quality or that are more appropriate to a local context, especially when applying the methods at the national or sub national levels.

The selection process resulted in the choice of the following source of data:

**The International boundaries dataset (IBD) **produced at 1:1,000,000 scale by the International and Administrative Boundaries Task Group [12] in the context of the United Nations Geographic Information Working Group (UNGIWG) was used in order to make the Atlas correspond to UN practice and to insure the connection with the freely accessible data from the Second Administrative Level Boundaries data set project (SALB) [13]. This data set is restricted and is not redistributed through the Atlas.

**The Road and hydrology network **extracted from the 1:1,000,000 scale Global Insight Plus dataset produced and distributed by Europa Technologies [14]. These data sets are licensed and are not redistributed through the Atlas.

**The Geology layer **acquired from four separate unprojected digital maps to cover the entire EM Region and the West Bank and Gaza Strip territory can be downloaded for free from the U.S. Geological Survey World Energy web site [15]. The four maps include: the 2002 Surficial Geology of Africa (1:5,000,000 scale), the 1998 Bedrock Geology of the Arabian Peninsula (1:2,000,000 and 1:5,000,000 scales), the 1999 Surficial Geology of the Islamic Republic of Iran (1:2,500,000 scale) and the 1998 Geologic Map of South Asia (1:5,000,000 and 1:10,000,000 scales).

**The soil type and soil texture **were derived from the 1974 FAO-UNESCO Soil Map of the World at a scale of 1:5,000,000 [16]. This data set is licensed and is not redistributed through the Atlas.

**The tectonic layer **has been extracted from the 1998 Digital Tectonic Activity Map (DTAM) created on the basis of several 30 meters resolution satellite images and 1:24'000 scale GIS databases by the United States National Aeronautics and Space Administration at the Goddard Space Flight Center [17]. This map shows the geologically and volcanically active features over the last one million years.

**The number of past flood events **was derived from flood polygons compiled by the Dartmouth Flood Observatory in their Global Active Archive of Large Flood Events for the period 1985 to 2005 [18].

**Elevation, slope, aspect and flow accumulation **was derived from the 30 arc seconds (1 km) resolution 2000 Shuttle Radar Topography Mission (SRTM) digital elevation model (DEM) [19].

**The land cover **was extracted from the 1998 1 km resolution global LandScan Land Cover Database [20] produced by the Oak Ridge National Laboratories and derived from the U.S. Geological Survey's (USGS) Global Land Cover Characteristics (GLCC) database [21]. This data set is licensed and is not redistributed through the Atlas.

**Figure 2:**
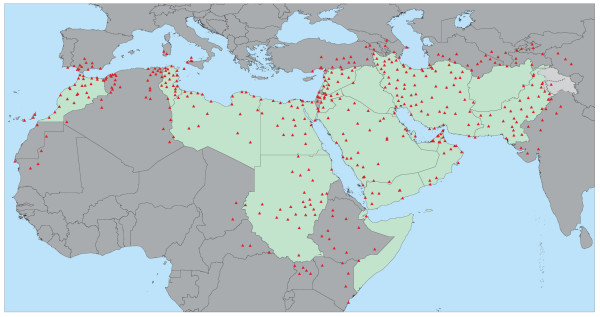
Location of the weather stations used for spatializing the distribution of the climatic variables.

**The location of the weather stations and associated climatic information **was obtained from the Global Surface Summary of the Day Data produced by the U.S. National Climatic Data Center (NCDC) [22]. 459 weather stations (Figure 2) are located in and around the EM Region and the West Bank and Gaza Strip territory but the climatic data are not necessarily complete for each of them which explain the differences in the numbers of stations reported in the method section of this paper. It is important to note that no data was available for Afghanistan, the Islamic Republic of Iran and Somalia and that no other source of information could be identified to fill these gaps. In order to support long-term planning by decision makers the daily maximum meteorological data have been calculated for a two, five and ten year return period. This results in the creation of three 3 different maps when spatializing the heat and wind speed hazards.

A document, directly accessible in the Atlas, describes the processes which lead to the compilation and homogenization of these layers. Specific metadata records for each of them are also available in the first volume of the Atlas.

## Methods and Results

A complete description of the literature review, causal factor selection process, the details on the models themselves and their application in ArcView ^© ^are reported in hazard specific methodology and implementation documents directly accessible in the first volume of the Atlas. This paper therefore only summarizes the review, selection process and application of these methods for each of the 5 selected hazards.

The resulting maps have been reclassified according to 5 relative levels of intensity (very low, low, medium, high and very high) comparable over the all EM Region and the West Bank and Gaza Strip territory.

If all the EM Region and the West Bank and Gaza Strip territory are covered in the Atlas, the results presented here only focus on a set of countries. Among these countries we find the Islamic Republic of Iran which, in the past century, had accounted for approximately 44 million people impacted (sum of injured, affected, homeless and killed) and estimated damages in excess of US$ 18 billion [23].

### Seismic hazard

Two types of methods are generally used to create seismic hazard maps:

• The deterministic approach which does not take local conditions (geology, or geotectonic characteristics (fault orientations)) into account, nor parameters such as the depth of the hypocenter and the distance to the epicenter, which represent an important limitation towards its use.

• The probabilistic methods based on peak ground acceleration (PGA) parameter. An advantage of these methods is that PGA is proportional to the force and is the most commonly mapped ground motion parameter. A second advantage of PGA-based models is that PGA is used as a reference for the construction of buildings which should be able to withstand an earthquake.

To develop the seismic hazard distribution map we used the data set developed by the Global Seismic Hazard Assessment Program (GSHAP) [24,25] for an exposure time of 50 years and a corresponding return period of 475 years, the resolution of which is one tenth of a degree resolution (around 12 kilometers).

For the Atlas the only modification to the GSHAP dataset was to reclassify the intensity of the peak ground acceleration into the five selected intensity levels as shown in Table 1.

**Table 1:** Correspondence between the GSHAP PGA ranges and the intensity levels of seismic hazard

**Peak ground acceleration (m/s^2^)**	**Intensity level**
0–0.2	Very low
0.2–0.8	Low
0.8–2.4	Medium
2.4–4	High
> 4	Very high

**Figure 3:**
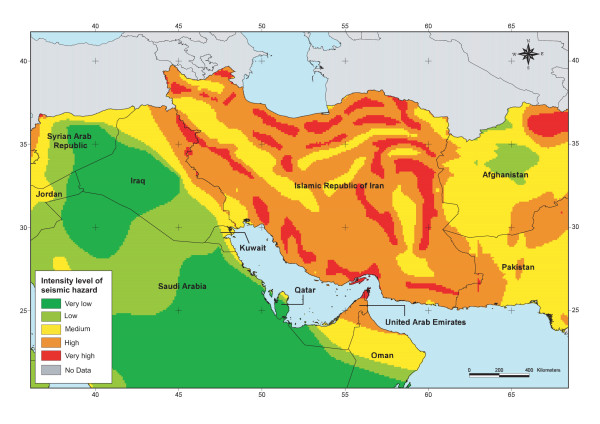
**Seismic hazard**. Spatial distribution of the intensity level of seismic hazard for part of the EM Region.

The map resulting from the application of this approach for part of the EM Region is shown in Figure 3.

In the first volume of the Atlas, the location and intensity of historically significant earthquakes for the past 2000 years, past geologic stress events, active volcanoes and the boundaries between tectonic plates are overlaid on the hazard distribution map in order to provide as much information as possible to the user.

### Heat hazard

The approach used for measuring the heat hazard has been to calculate the spatial distribution of the annual daily maximum heat index (HI) for the all EM Region and the West Bank and Gaza Strip territory.

The heat index (HI) sometimes also referred to as the "apparent temperature" is defined by the American Red Cross [26] as a "prolonged period of excessive heat and humidity". Nevertheless, and according to Ken Granger and Michael Berechree [27], the use of apparent temperature for an individual day can assist in the evaluation of heat. This is the approach used in the present work.

This index, given in degrees Fahrenheit (*F*), is a measure of how hot it feels when relative humidity (*rh*) is added to the actual air temperature. Indeed when humidity is low, the apparent temperature will be lower than the air temperature, since perspiration evaporates rapidly to cool the body. However, when humidity is high (i.e. the air is saturated with water vapour) the apparent temperature "feels" higher than the actual air temperature, because perspiration evaporates more slowly.

In the Atlas, the maximum heat index is calculated using Steadman's formula [28,29]. This formula, presented here, produces approximations for the heat index with an error of ± 1.3°F.:

*Index*_*heat *_= -42.379 + (2.04901523 × *T*) + (10.14333127 × *rh*)

-(0.22475541 × *T *× *rh*) - (6.83783 × 10^-3 ^× *T*^2^)

-(5.481717 × 10^-2 ^× *rh*^2^) + (1.22874 × 10^-3 ^× *T*^2 ^× *rh*)

+(8.5282 × 10^-4 ^× *T *× *rh*^2^) - (1.99 × 10^-6 ^× *T*^2 ^× *rh*^2^)

Where:

*T *= maximum air temperature (degrees Fahrenheit)

*rh *= maximum relative humidity (percent)

This equation, which is derived from Steadman's table [30], is only useful for temperatures above 80°F and a relative humidity above 40%.

**Figure 4:**
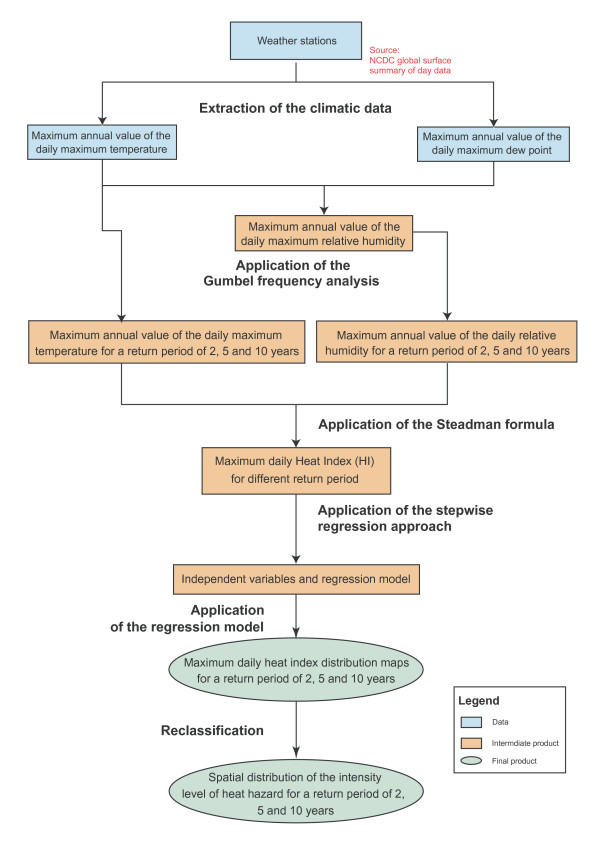
Methodology for the generation of heat hazard distribution maps.

For the Atlas the climatic data were obtained from weather stations located in and around the EM Region and the West Bank and Gaza Strip territory (see the data section) on which a statistical frequency analysis was conducted to estimate the annual daily maximum heat indices for different return periods. Finally, a multiple regression analysis was used to produce an interpolation surface for the entire EM Region and the West Bank and Gaza Strip territory. The methodology is outlined in Figure 4 and described in the following sections.

### Extraction of the climatic data

The daily maximum temperature and the daily maximum dew point temperatures spanning over at least 7 years during the period 1994–2005 for 453 of the 459 weather stations located in and around the EM Region and the West Bank and Gaza Strip territory, were extracted from the global surface summary of day dataset produced by the National Climatic Data Center (NCDC) [31].

For each station, the daily maximum relative humidity was obtained using the following formula [29]:

rh=ees×100
 MathType@MTEF@5@5@+=feaafiart1ev1aaatCvAUfKttLearuWrP9MDH5MBPbIqV92AaeXatLxBI9gBaebbnrfifHhDYfgasaacH8akY=wiFfYdH8Gipec8Eeeu0xXdbba9frFj0=OqFfea0dXdd9vqai=hGuQ8kuc9pgc9s8qqaq=dirpe0xb9q8qiLsFr0=vr0=vr0dc8meaabaqaciaacaGaaeqabaqabeGadaaakeaacqWGYbGCcqWGObaAcqGH9aqpdaWcaaqaaiabdwgaLbqaaiabdwgaLnaaBaaaleaacqWGZbWCaeqaaaaakiabgEna0kabigdaXiabicdaWiabicdaWaaa@39B6@

Where:

*e*: actual vapor pressure e=6.11×107.5×Td237.7+Td
 MathType@MTEF@5@5@+=feaafiart1ev1aaatCvAUfKttLearuWrP9MDH5MBPbIqV92AaeXatLxBI9gBaebbnrfifHhDYfgasaacH8akY=wiFfYdH8Gipec8Eeeu0xXdbba9frFj0=OqFfea0dXdd9vqai=hGuQ8kuc9pgc9s8qqaq=dirpe0xb9q8qiLsFr0=vr0=vr0dc8meaabaqaciaacaGaaeqabaqabeGadaaakeaacqWGLbqzcqGH9aqpcqaI2aGncqGGUaGlcqaIXaqmcqaIXaqmcqGHxdaTcqaIXaqmcqaIWaamdaahaaWcbeqaamaaleaameaacqaI3aWncqGGUaGlcqaI1aqncqGHxdaTcqWGubavdaWgaaqaaiabdsgaKbqabaaabaGaeGOmaiJaeG4mamJaeG4naCJaeiOla4IaeG4naCJaey4kaSIaemivaq1aaSbaaeaacqWGKbazaeqaaaaaaaaaaa@46DB@

*e*_*s*_: saturated vapor pressure es=6.11×107.5×T237.7+T
 MathType@MTEF@5@5@+=feaafiart1ev1aaatCvAUfKttLearuWrP9MDH5MBPbIqV92AaeXatLxBI9gBaebbnrfifHhDYfgasaacH8akY=wiFfYdH8Gipec8Eeeu0xXdbba9frFj0=OqFfea0dXdd9vqai=hGuQ8kuc9pgc9s8qqaq=dirpe0xb9q8qiLsFr0=vr0=vr0dc8meaabaqaciaacaGaaeqabaqabeGadaaakeaacqWGLbqzdaWgaaWcbaGaem4Camhabeaakiabg2da9iabiAda2iabc6caUiabigdaXiabigdaXiabgEna0kabigdaXiabicdaWmaaCaaaleqabaWaaSqaaWqaaiabiEda3iabc6caUiabiwda1iabgEna0kabdsfaubqaaiabikdaYiabiodaZiabiEda3iabc6caUiabiEda3iabgUcaRiabdsfaubaaaaaaaa@459C@

Where:

*T *= air temperature degrees Celsius

*T*_*d *_= dewpoint temperature degrees Celsius

### Estimation of the maximum meteorological data for different return periods

In order to facilitate decision-makers' long range planning, the daily maximum meteorological data were calculated for a two, five and ten year return period. This was done using a probability distribution function [32-34] which examines the relationship between the past magnitude and frequency of occurrence of the phenomena to identify some statistical regularity between them and then extrapolate the values into the future.

Despite the extensive literature on the topic, there is no preferred distribution function for the frequency analysis of meteorological data because each function has a unique set of advantages and disadvantages. In addition, the problem is compounded when evaluating meteorological data for return periods that exceed the length of the observed record.

The Gumbel extreme value distribution function [35-38] was finally selected for the Atlas among the multitude of distribution functions used when analysing hydrologic and meteorological data [39]. This function reads as follow [40]:

F(x)=e−e−x−ab
 MathType@MTEF@5@5@+=feaafiart1ev1aaatCvAUfKttLearuWrP9MDH5MBPbIqV92AaeXatLxBI9gBaebbnrfifHhDYfgasaacH8akY=wiFfYdH8Gipec8Eeeu0xXdbba9frFj0=OqFfea0dXdd9vqai=hGuQ8kuc9pgc9s8qqaq=dirpe0xb9q8qiLsFr0=vr0=vr0dc8meaabaqaciaacaGaaeqabaqabeGadaaakeaacqqGgbGrcqqGOaakcqWG4baEcqqGPaqkcqGH9aqpcqqGLbqzdaahaaWcbeqaaiabgkHiTiabbwgaLnaaCaaameqabaGaeyOeI0YaaSaaaeaacqqG4baEcqGHsislcqqGHbqyaeaacqqGIbGyaaaaaaaaaaa@3BCD@

where:

F(*x*): Cumulative distribution function

*a *and *b*: Adjustment parameters with *a *being a location and *b *a scale parameter.

Replacing x−ab
 MathType@MTEF@5@5@+=feaafiart1ev1aaatCvAUfKttLearuWrP9MDH5MBPbIqV92AaeXatLxBI9gBaebbnrfifHhDYfgasaacH8akY=wiFfYdH8Gipec8Eeeu0xXdbba9frFj0=OqFfea0dXdd9vqai=hGuQ8kuc9pgc9s8qqaq=dirpe0xb9q8qiLsFr0=vr0=vr0dc8meaabaqaciaacaGaaeqabaqabeGadaaakeaadaWcaaqaaiabdIha4jabgkHiTiabdggaHbqaaiabdkgaIbaaaaa@31BA@ with the reduced variate, *u*, the cumulative distribution function becomes,

F(*x*) = *e*^-υ^

*u *= -ln[-ln F(*x*)] = -ln[-ln (1-1/*T*)]

Where *T *= Return period

For each of the 453 weather stations, the result of this operation was a measure of the annual daily maximum temperature and relative humidity for a two, five and ten year return period.

The annual daily maximum heat index was then calculated for each weather station using Steadman's formula [28,29] reported in the introduction of this section.

### Spatialization of the annual daily maximum heat index

Spatial interpolation is widely used for translating irregularly scattered meteorological data (data collected at discrete locations (i.e. at points)) into continuous data surfaces (rasters).

The choice of interpolation method is especially important in the EM Region and over the West Bank and Gaza Strip where meteorological data are sparse and large value changes can be observed over short spatial distances. Additionally, the spatial density, distribution and spatial variability of sampling stations influence the choice of the interpolation technique [41].

Given a set of meteorological data, researchers are confronted with a variety of stochastic and deterministic spatial interpolation methods to estimate meteorological data values at un-sampled locations, namely:

• **Deterministic **estimation methods which include the **inverse distance weighting **[42-44] and the **Spline method **[42,43,45].

• **Stochastic **methods which include the **kriging and cokriging **[36,43,46,47] as well as the **polynomial regression **[36,48,49].

For a summary description of these spatial interpolation methods please refer to Collins and Bolstad [43] and El Morjani, [36].

Unfortunately, the data characteristics observed in the EM Region and the West Bank and Gaza Strip territory (low spatial data density, high spatial variability and the absence of meteorological data for some countries) resulted in implausible outputs from the application of the above mentioned methods, more specifically:

- The application of the inverse distance weighting method across a test area (Afghanistan, Islamic Republic of Iran and Pakistan) showed specking or "birds eye" effects around the station locations which is not correct as the spatial variation did not follow a regular trend.

- The application of the kriging technique over the same test area produced results inconsistent with the original data. None of the candidate models used was able to fit to the statistical cross validation to the spatial semivariogram. (spherical, exponential, or Gaussian) and could not be set up in a kriging model. This may indicate that the density of weather stations is too low and the area too large to support the use of kriging.

It has therefore been necessary to find another model that would produce the best estimation possible when generating a continuous surface for the annual daily maximum heat index.

A literature review was carried out to first identify the following set of variables that are significantly correlated to the heat index:

• **Topographical factors **such as elevation (*Z*); the mean elevation within a 9 pixel window (*Z9*); aspect (*Asp*) and slope (*Slp*);

• **Geographical factors **such as relative longitude (*X*) and relative latitude (*Y*); and the distance to the nearest coastline (*d_Coast*).

Since the topographical factors of each weather station were already part of the datasets being prepared for the Atlas, only the distance to the nearest coastline, the relative latitude and longitude of each station have been measured.

Given the number of identified variables to which their square and cube have also been added, the stepwise (back and forth steps) linear regression technique was then used to identify the statistical significance of the above listed independent parameters and their relative contribution to the determination of the dependent variable (the heat index) thereby eliminating any insignificant variables [36].

Because of their different climatologic characteristics, it was decided to divide the study area into the following four zones in order to perform this analysis:

• Zone 1: Morocco

• Zone 2: Djibouti, Egypt, Libyan Arab Jamahiriya, Somalia, Sudan, Tunisia.

• Zone 3: Bahrain, Iraq, Jordan, Kuwait, Lebanon, Oman, Qatar, Saudi Arabia, Syrian Arab Republic, United Arab Emirates, West Bank and Gaza Strip and Yemen

• Zone 4: Afghanistan, Islamic Republic of Iran, Pakistan.

This meant that a stepwise linear regressions analysis had to be performed separately for each of these zones and return period.

As an example, the result of the stepwise regression analysis performed for the 49 meteorological stations located in Zone 1 for a two year return period is shown in Table 2.

**Table 2:** Heat hazard

**Variable**	**Regression coefficient**	**Standard error**	**t-statistic value**	**Signification probability >|t|**
(Intercept)	90.267436358174	5.6869	8.4465	0
Dist. to the nearest coastline	0.648406525194	0.1012	6.4058	0.0000
(Dist. to the nearest coastline)^2^	-0.002046701602	0.0005	-3.7318	0.0005
(Dist. to the nearest coastline)^3^	0.000002105930	0.0000	2.7802	0.0081
Latitude	0.043177051421	0.0190	2.2712	0.0284
(Latitude)^2^	-0.000020781161	0.0000	-2.3431	0.0240
(Mean Elevation)^2^	-0.000040550952	0.0000	-3.7478	0.0005
(Mean Elevation)^3^	0.000000009778	0.0000	2.5671	0.0139
				
Residual standard error	3.1887580414			
degrees of freedom	41			
Multiple R^2^	0.923138021554			
F-statistic	27.259883813			
Probability (F_statistic)	0.0000			

Result of the stepwise regression analysis for Zone 1 and a two year return period

This analysis reveals that only three of the seven independent variables contributed to explain 92% of the variance in the annual daily maximum heat index for a two year return period.

These variables are combined according to the following equation:

HI = (0.648406525194 × d_Coast) - (0.002046701602 × d_Coast ^2) + (0.000002105930 × d_Coast ^3) + (0.043177051421 × Y) - (0.000020781161 × Y^2) - (0.000040550952 × Z^2) + (0.000000009778 × Z^3) + 90.267436358174

This model can be considered as valid and reliable in view of the strong correlation that exist between the heat and the variables (R = 0.96) and with a high degree of confidence in the selected variables in view of the very low value obtained for the Probability (F_statistic). Similar results have been obtained for the other zones and/or return periods (correlation coefficient, R, varying between 0.80 and 0.92).

Once the regression models attached to each zone and return period were identified, they were applied to the existing data set before aggregating them to obtain the distribution of the maximum heat index over the all EM Region and the West Bank and Gaza Strip territory.

### Creation of the heat hazard distribution map

**Figure 5:**
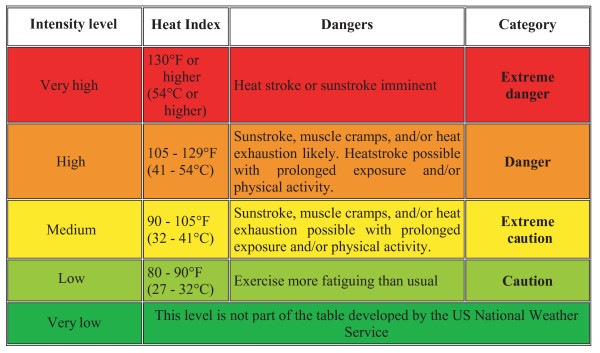
Correspondence between the intensity level of heat hazard and the US National Weather Service classification.

The spatial distribution of heat hazard was derived from the annual daily maximum heat index distribution maps by reclassifying them into five intensity levels on the basis of the table developed by the US National Weather Service [29] which is shown in Figure 5. This table allows the correspondence between the intensity levels of heat hazard and the US National Weather Service classification which is made of heat index ranges and heat induced physiological effects (dangers and category).

**Figure 6:**
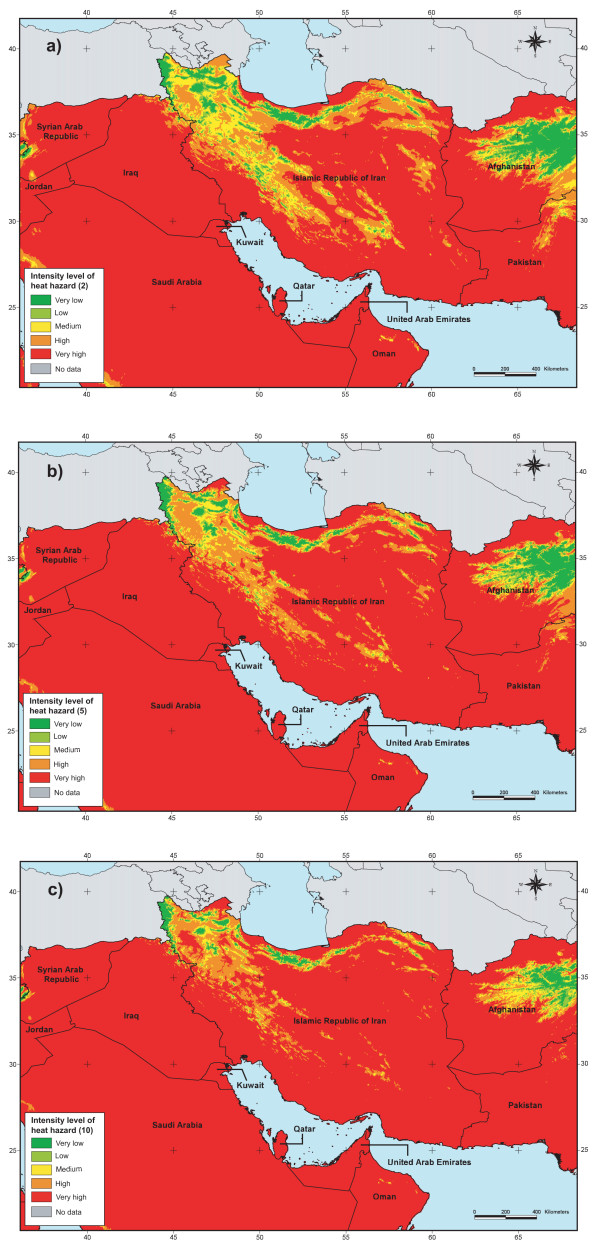
**Heat hazard**. Spatial distribution of the intensity level of heat hazard for part of the EM Region for a 2 (a), 5 (b) and 10 (c) year return period.

The result obtained through the application of this approach is presented in Figure 6 for part of the EM Region.

### Wind speed hazard

The process used to generate the wind speed hazard intensity level distribution map for different return periods (two, five and ten years) was very similar to the one used for the heat hazard, the main difference being the climatic factor that was spatialized and the fact that the wind speed was directly obtained for each weather station without needing to apply a particular formula. In brief the following steps were applied:

1. The annual maximum daily mean wind speed for 458 of the 459 weather stations (Figure 2) which climatic records were spanning at least over 7 years was extracted from the global surface summary of day data set produced by the National Climatic Data Center (NCDC) for the period of 1994 to 2005 [31].

2. For each station, the Gumbel frequency analysis was used to estimate the annual maximum daily mean wind speed values for the three selected return periods.

3. The relevant variables for each zone and return period were selected among the same variables than the ones listed for the heat hazard using the stepwise regression method.

4. The resulting regressions, which present a correlation coefficient, R, varying between 0.78 and 0.97, were used to spatialize the distribution of the annual maximum daily mean wind speed for each return period and zone.

5. The resulting annual maximum daily mean wind speed distribution maps were reclassified to obtain the spatial distribution of the intensity level of wind speed hazard s based on a modified Beaufort classification used by the US National Weather Service [50] as reported in Table 3.

**Table 3:** Correspondence between the wind speed ranges and the intensity level of wind speed hazard

**Wind speed ranges**	**Intensity level**
< 3.3 m/s	Very low
3.3 – 10.7 m/s	Low
10.7 – 17.1 m/s	Medium
17.1 – 24.4 m/s	High
> 24.4 m/s	Very high

**Figure 7:**
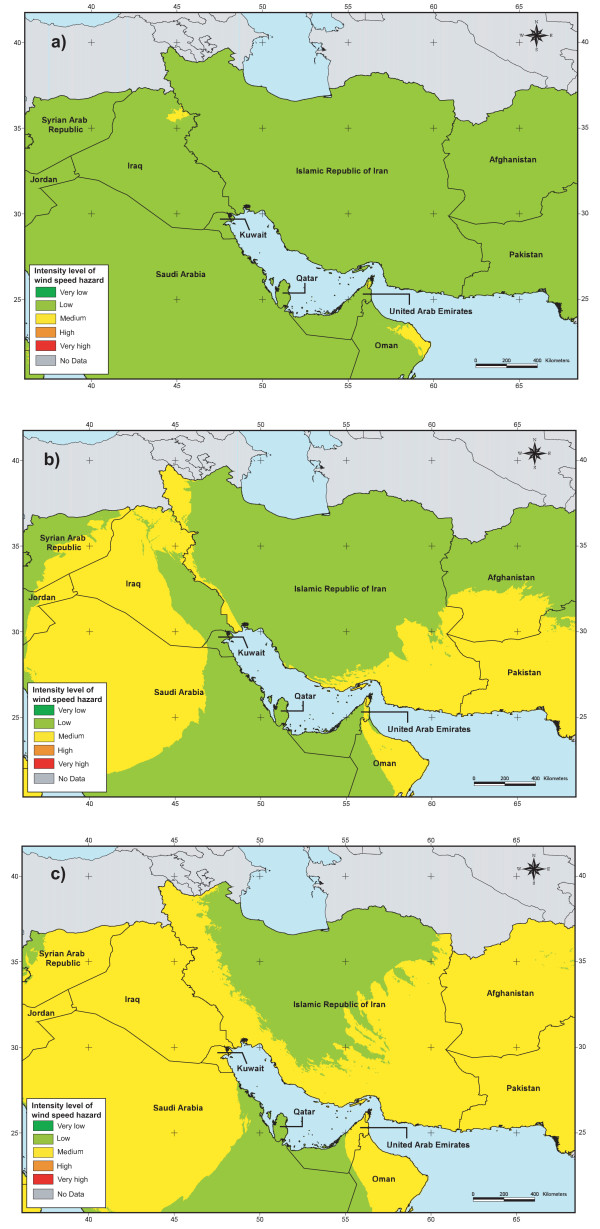
**Wind speed hazard**. Spatial distribution of the intensity level of wind speed hazard for part of the EM Region for a 2 (a), 5 (b) and 10 (c) year return period.

The result obtained through the application of this approach is presented in Figure 7 for part of the EM Region.

### Flood hazard

Over the past 20 years a large amount of research has been conducted to identify techniques for the generation of flood hazard distribution maps. These techniques include hydrologic frequency analysis, hydraulic modelling, hydrological modelling and statistical methods.

The statistical technique was finally chosen here first because the flood frequency analysis was not practical for the purposes of the Atlas for two reasons: 1)flood frequency analysis relies on historical meteorological and stream flow data that are influenced by changes in stream and flood flows themselves caused by reservoir regulation, channel improvements (levees) and land use changes. It is in fact difficult to develop a long range model that accounts for all of these flow changes, 2) these data are not readily available for the EM Region and the West Bank and Gaza Strip territory.

Second, the hydraulic models (i.e. Hydrologic Engineering Center's River Analysis System (HEC-RAS) model) could not be applied in the EM Region and the West Bank and Gaza Strip territory because important input data could not be acquired.

Finally, the hydrologic models were not viable because they would have required careful and accurate calibration over the all EM Region and the West Bank and Gaza Strip territory to yield accurate estimates of flood prone areas which was beyond the scope and resources of the project.

Among the different statistical techniques available, the one selected was based on the work done by Islam and Sado [51] in Bangladesh. This statistical technique presented several advantages:

• It yields realistic estimates without using an empirical model.

• It requires historical flood distribution and causal factor data which are readily available for the entire EM Region and the West Bank and Gaza Strip territory.

• It can be readily applied with the GIS technologies used throughout the development of the Atlas.

• It considers both the susceptibility of each area to inundation and factors related to flood emergency management.

This technique combines historic flood frequency data with flood causal factor distribution to enable the calculation of a weighted score for each of these factors. The weighted scores are then aggregated to generate a flood hazard index (FHI) which is used to map the distribution of flood hazard.

Having decided to use this particular technique, the following causal factors were selected based on a review of different case studies reported in the literature and their relevance to the EM Region and the West Bank and Gaza Strip territory:

• **Land use and land cover: **influence a number of parameters in the hydrologic cycle including interception, infiltration, concentration, and runoff behaviours. Together these characteristics yield information about the hydrologic response and the degree of flood hazard [39,51-53].

• **Elevation**: the likelihood of a flood increases as the elevation of a location decreases [2,53,54].

• **Soil type and texture**: both play a role in determining the water holding and infiltration characteristics of an area, and consequently in the probability of flood to occur [52,53].

• **Lithology**: floods are more likely to occur in areas that consist of largely impermeable surface geology [51]. However, since lithology data is not available for the EM Region and the West Bank and Gaza Strip territory, surface geology has been used instead.

• **Flow accumulation volume and distance from the flow accumulation path: **have been combined into one in the context of the present work based on the fact that areas located close to the flow accumulation path, and in particular when a large volume has been accumulated upstream, are more likely to get flooded [7,51,52].

• **Precipitation**: the likelihood of a flood increases as the amount of rain and snow at a location increases [52,53]. In this study, we used the annual daily maximum precipitation with a five year return period calculated using the same approach as the one followed for the spatilization of the annual maximum daily mean wind speed.

In order to integrate the probability component in this work and therefore obtain the distribution of the hazard, the spatial distribution of these causal factors has been crossed with the spatial distribution of the number of past flood events observed for the EM Region and the West Bank and Gaza Strip territory over the 1985–2005 period.

**Figure 8:**
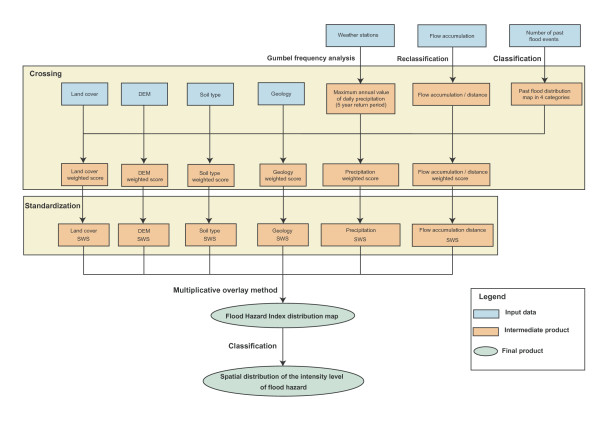
Methodology for generating the spatial distribution of the intensity level of flood hazard.

Once identified and homogenized (see the data section of this paper) the geographic distribution of these causal factors and of the number of past flood events were combined using the process illustrated in Figure 8 to generate the spatial distribution of the intensity level of flood hazard. This process, which is detailed in the coming sections, was applied simultaneously to the entire EM Region and the West Bank and Gaza Strip territory.

### Classification of the number of past flood events distribution map

The spatial distribution of the number of previous flood events observed between 1985 and 2005, which can be considered as a measure of the flood frequency, was classified into four specific levels of historical hazard (Table 4).

**Table 4:** Correspondence between the number of previous floods and the historical flood hazard level

**Number of previous flood events 1985–2005**	**Historical flood hazard level**
0	None
1 – 3	Low
4 – 7	Medium
> 7	High

Similarly, the other causal factors were reclassified into ordinal classes following the process reported in the "Methodology and procedures for modelling the spatial distribution of flood hazard" document available in the first volume of the Atlas.

### Estimation of the weighted scores and creation of the flood hazard index (FHI)

By crossing the reclassified flood frequency distribution map with each causal factor distribution grid we obtain the area percentage distribution of each class/category according to the historical flood hazard classification reported in Table 4.

As an example, Table 5 shows the distribution obtained for the different land cover categories observed in the EM Region and the West Bank and Gaza Strip territory.

**Table 5:** Flood hazard

	**Historical flood hazard level**					
			
**Land cover category**	**None (%)**	**Low (%)**	**Medium (%)**	**High (%)**	**Weighted score**	**SWS**
Built areas	7.741	51.817	26.540	13.902	200.948	**3**
Dry cropland and pasture	6.103	38.959	37.281	17.657	239.089	**3**
Cropland/grassland	19.716	67.139	12.639	0.506	107.586	**2**
Cropland/woodland	23.442	59.492	16.867	0.200	111.092	**2**
Shrubland	17.921	68.436	12.834	0.808	110.980	**2**
Shrubland/grassland	10.323	86.129	3.548	0.000	96.774	**2**
Savanna	20.857	62.403	15.573	1.168	114.961	**2**
Deciduous, evergreen forest	28.686	52.083	18.109	1.122	112.019	**2**
Water	4.926	57.475	30.747	6.852	183.975	**2**
Herbaceous wetland	46.667	33.333	20.000	0.000	93.333	**1**
Wooded wetland	4.762	61.905	33.333	0.000	161.905	**2**
Barren	21.883	73.357	4.727	0.033	87.703	**1**
Wooded tundra	27.121	48.485	24.394	0.000	121.667	**2**
Mixed tundra	7.143	78.571	14.286	0.000	121.429	**2**
Snow or ice	64.834	30.246	4.920	0.000	45.007	**1**
Partly developed	10.759	59.034	22.069	8.138	165.931	**2**
Unclassified	21.277	63.830	14.894	0.000	108.511	**2**

Area percentage distribution for the different historical flood hazard levels, weighted score and standardized weighted scores (SWS) for the different land cover categories observed in the study area

The weighted score for each category was then calculated as the sum of the products between the area percentage of the category for each historical flood hazard level and the associated damage coefficient using the following equation [51]:

Weighted score = (0 × *A*) + (1 × *B*) + (3 × *C*) + (5 × *D*)

Where:

*A *= area percentage of the category in areas with no historical flood hazard

*B *= area percentage of the category in areas with low historical flood hazard

*C *= area percentage of the category in areas with medium historical flood hazard

*D *= area percentage of the category in areas with high historical flood hazard

0,1,3,5 = corresponding damage coefficients attached to each specific historical flood hazard class to express the severity of each level of historical flood hazard

As an example, Table 5 shows the resulting weighted score obtained for each landcover class observed in the EM Region and the West Bank and Gaza Strip territory. Basically, the more frequently a category is found in areas with high historical flood hazard, the higher the weighted score.

Once the weighted scores for all of a factor's categories has been calculated they were standardized and rescaled into ordinal classes according to a scale going from 1 to 3, 1 indicating categories with the lowest likelihood for a flood to occur in this area, and 3 the categories with the highest.

Finally, a linear interpolation scheme was used to standardize the weighted scores of all the causal factors to allow them to be comparable across factors. As an example, Table 5 presents the standardized weighted scores (SWS) obtained for the land cover categories throughout the EM Region and the West Bank and Gaza Strip territory.

### Creation of the flood hazard distribution map

Each causal factor distribution map was reclassified to contain the distribution of the corresponding standardized weighted score before being combined using the multiplicative overlay method. For each cell, this method multiplies the score of all the causal factors together to produce the spatial distribution of the flood hazard index (FHI).

Finally, the flood hazard index (FHI) distribution map was reclassified into the five intensity levels using a natural breaks scheme to derive the flood hazard distribution map.

**Figure 9:**
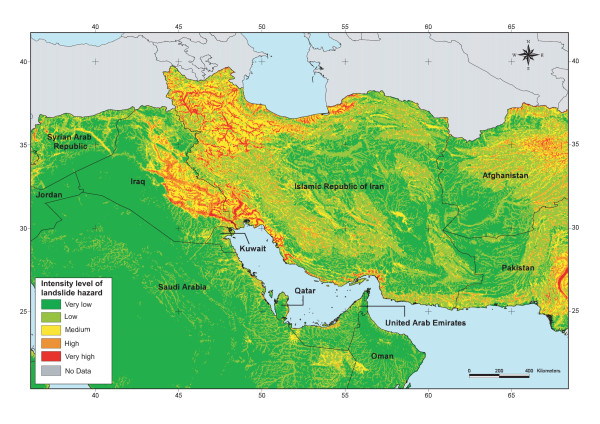
**Flood hazard**. Spatial distribution of the intensity level of flood hazard for part of the EM Region.

The result obtained through the application of this approach for part of the EM Region is presented in Figure 9.

### Landslides

Like other hazards, many techniques have been proposed in the literature for the evaluation and zonation of landslide hazards. Broadly, the techniques fall into two types: qualitative, or direct, techniques and quantitative, or indirect, techniques.

Qualitative techniques involve direct observation and assessment of the study area while quantitative techniques include deterministic, statistical or probabilistic models [55,56]. Even if there is no consensus among researchers on the best or most appropriate technique to be used [57,58], the ones that do include a frequency or probability component should be used if we want to talk about hazard (see the definition in the introduction). Unfortunately it was not possible to use one of these techniques because qualitative techniques are time-consuming, expensive and extremely labour intensive and because statistical, or probabilistic models need a comprehensive landslide inventory which was not available for the countries in the EM Region and the West Bank and Gaza Strip territory.

A deterministic model, based on prior knowledge of the physical causal factors that influence the likelihood of landslide occurrence, was therefore used. In this method, the causal factors are mapped, ranked and weighted based on their assumed or expected importance in mass wasting processes [5,59,60]. Then the causal factors are combined into a single surface which quantifies the likelihood of a mass wasting event to occur in a specific geospatial location.

The results from the application of this method provide the distribution of landslide susceptibility. Despite this limitation these results were used as a first approximation of the landslide hazard distribution.

The literature review identified nine causal factors of relevance to the EM Region and the West Bank and Gaza Strip territory:

• **Slope**: the likelihood of a landslide increases as slope increases [6,61,62].

• **Relative elevation: **the likelihood of a landslide increases as the relative elevation of a location increases [4-6].

• **Precipitation: **can first destabilize rock formations and secondly increases the amount of water in surface soils [4,5]. As with flood hazard the annual daily maximum precipitation with a five year return period has been used.

• **Land use and land cover: **play a critical role in slope stabilization through different mechanisms taking place at the level of the vegetation root systems and the foliage (interception). Additionally, areas with little or no vegetative land cover and degraded areas are predisposed to landslides and mass wasting events [4-6,61].

• **Distance from roads: **the construction of roads that traverse slopes can destabilize an area by cutting into the slope, removing lateral support and/or steepening a slope [4-6].

• **Distance from geologic faults: **the likelihood of seismic shocks or earthquakes triggering a mass wasting event increases as the distance to the fault decreases. [4-6,61].

• **Distance from drainage networks: **proximity to hydrologic features such as streams, rivers or oceans can decrease slope stability by eroding the foot of a slope, saturating a slope, increasing the soil's pore water pressure, and by steepening slopes. This effect is reinforced during periods of high precipitation [5,6].

• **Soil texture: **the internal cohesion and friction of soil greatly influences its capacity to move [5,6,62].

• **Lithology: **Many sources identify the surface lithology as a very important factor that can predispose an area to landslides and mass wasting events [5,63]. Unfortunately, the lack of information regarding the distribution of the lithology over the EM Region and the West Bank and Gaza Strip territory did not allow us to include this causal factor in the model.

**Figure 10:**
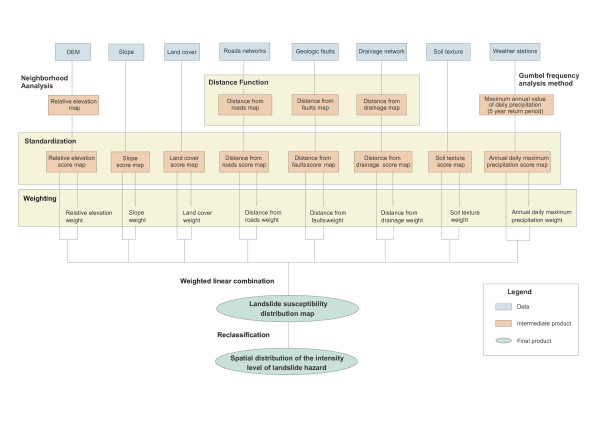
Methodology for the generation of the spatial distribution of the intensity level of landslide hazards.

Once extracted or derived from the data at disposal, the geographic distributions of these factors were simultaneously combined for the entire EM Region and the West Bank and Gaza Strip territory using the process reported in Figure 10. These steps are described in more detail in the coming sections.

### Standardization of the causal factors

To integrate both the continuous and discrete aspects of the causal factors into the multi-criteria analysis it was necessary to standardize them on a scale from 0 to 10, 0 being attributed to areas where landslides were least likely to occur and 10 areas where the likelihood of such events was high.

Linear scaling was used to standardize the continuous factors (distance from hydrologic features, roads and faults, relative elevation, annual daily maximum precipitation and slope) into nominal classes using the following equation [64,65]:

xi=(Ri−Rmin⁡)(Rmax⁡−Rmin⁡)×standardized range
 MathType@MTEF@5@5@+=feaafiart1ev1aaatCvAUfKttLearuWrP9MDH5MBPbIqV92AaeXatLxBI9gBaebbnrfifHhDYfgasaacH8akY=wiFfYdH8Gipec8Eeeu0xXdbba9frFj0=OqFfea0dXdd9vqai=hGuQ8kuc9pgc9s8qqaq=dirpe0xb9q8qiLsFr0=vr0=vr0dc8meaabaqaciaacaGaaeqabaqabeGadaaakeaacqWG4baEdaWgaaWcbaGaemyAaKgabeaakiabg2da9maalaaabaGaeiikaGIaemOuai1aaSbaaSqaaiabdMgaPbqabaGccqGHsislcqWGsbGudaWgaaWcbaGagiyBa0MaeiyAaKMaeiOBa4gabeaakiabcMcaPaqaaiabcIcaOiabdkfasnaaBaaaleaacyGGTbqBcqGGHbqycqGG4baEaeqaaOGaeyOeI0IaemOuai1aaSbaaSqaaiGbc2gaTjabcMgaPjabc6gaUbqabaGccqGGPaqkaaGaey41aqRaee4CamNaeeiDaqNaeeyyaeMaeeOBa4MaeeizaqMaeeyyaeMaeeOCaiNaeeizaqMaeeyAaKMaeeOEaONaeeyzauMaeeizaqMaeeiiaaIaeeOCaiNaeeyyaeMaeeOBa4Maee4zaCMaeeyzaugaaa@6344@

Where *x*_*i *_= the standardized value assigned to a pixel presenting a raw value of *R*_*i*_

This equation uses the minimum and maximum values (R_*min *_and R_*max*_) as scaling points, the standardized range being the range of the values of the ordinal classes (10 in this case).

When the correlation between the causal factor and landslide is positive, meaning that the likelihood of a landslide increases as the value of the causal factor increases (slope, relative elevation, and annual daily maximum precipitation), *R*_min _is equal to the lowest raw value in the data set and *R*_max _to the highest one.

When the correlation between the causal factor and landslide is negative meaning that the likelihood of a landslide increases as the value of the causal factor decreases (distance from hydrologic features, roads and faults), then *R*_min _is equal to the highest raw value in the data set and *R*_max _to the lowest one.

For land cover, the original classification was aggregated into simplified classes based on vegetation density. These new classes were then ranked according to the likelihood that a landslide could occur (the lower the density of vegetation the higher the likelihood [66,67]) and an ordinal class from 1 to 10 was attributed to each.

The same process was followed for soil texture, the original classes being aggregated based on their hydraulic properties and their likelihood of triggering landslides or mass wasting events [68].

### Weighting of the causal factors

The causal factors used in this analysis do not present the same level of influence when it comes to measuring their potential to triggering a landslide in an area. To account for these differences in significance, weights were assigned to each of the factors so that, statistically, the more influential the factor the higher the weight.

There are numerous techniques that can be used for weighting factors in a multi-criteria analysis [69]. This work uses the pairwise comparison method developed in the context of the Analytical Hierarchy Process (AHP) [70] because it allows for the development and prioritization of factor weights based upon quantitative input from numerous individuals.

In a pairwise comparison, each factor is ranked verbally by importance, using comparisons criteria such as "more important" or "strongly more important"; and then converted to a first scale of one to nine. The preferences are then normalized to a second scale of 0 to 1 to create weights. In the case of landslide a group of three structural geologists, two hydrogeologists, one geologist and one environmental scientist were consulted individually to determine the weights to be attached to each causal factor. The weights obtained from each member of the group were then combined in order to assign a single weight for each factor (Table 6).

**Table 6:** Causal factor weights resulting from the pairwise comparison exercise for landslide hazard

**Priority**	**Factor**	**Weight**
1	Slope	0.207
2	Soil texture	0.188
3	Annual daily maximum precipitation (5 years)	0.144
4	Distance from faults	0.138
5	Relative elevation	0.105
6	Distance from drainage networks	0.097
7	Distance from roads	0.063
8	Land use	0.058
	Sum	1.000

### Creation of the landslide susceptibility and hazard distribution map

To generate the landslide susceptibility distribution map the standardized causal factor distribution maps were combined using the weighted linear combination (WLC) method. This method multiplies each map by its corresponding weight before adding the results together to obtain the distribution of the landslide susceptibility index (LSI). This can be illustrated by the following formula:

*LSI *= (*S *× *W*_*S*_) + (*ST *× *W*_*ST*_) + (*P *× *W*_*P*_) + (*DF *× *W*_*DF*_) + (*E *× *W*_*E*_) + (*DDN *× *W*_*DDN*_) + (*DR *× *W*_*DR*_) + (*L *× *W*_*L*_)

Where:

*S *= slope

*ST *= soil texture

*P *= annual daily maximum precipitation (5 years)

*DF *= distance from faults

*E *= relative elevation

*DDN *= distance from the drainage network

*DR *= distance from roads

*L *= land use

*W *= causal factor weight

**Figure 11:**
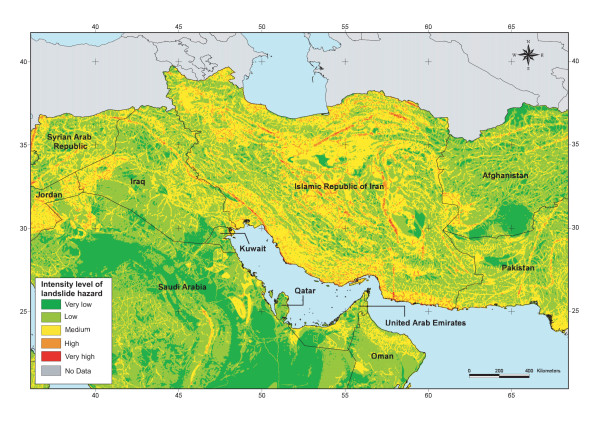
**Landslide hazard**. Spatial distribution of the intensity level of landslide susceptibility/hazard for part of the EM Region.

The map resulting from the application of this formula was then reclassified into the five levels of intensity using the linear scaling technique. As mentioned earlier the reclassified map, partly presented in Figure 11, is used as an approximation of the spatial distribution of the intensity level of landslide hazard over the EM Region and the West Bank and Gaza Strip territory.

### Multi hazard

Considering each hazard separately is a good approach for looking at specific health issues that might be linked to each of them. However, it is also important to identify potential hotspots where the population might be exposed to several hazards at the same time.

To get the spatial distribution of the intensity level of multihazard, weights were assigned in a first stage to each of the 5 hazards on the basis of the human (numbers of people killed, injured, homeless, and affected) and economic (total damage expressed in US$) impact of each event reported in the Emergency Disasters Data Base (EM-DAT) coming from the Center for Research on the Epidemiology of Disasters (CRED) [71]. For each hazard, the regional averages of these indicators have been computed, standardized, weighted using the pairwise comparison technique and aggregated using the weighted linear combination technique (WLC).

Table 7 shows the normalized weights which resulted from the application of these steps.

**Table 7:** Normalized weights applied to the different hazards when calculating multihazard

Hazard	Normalised weight
Seismic	0.41
Flood	0.36
Wind speed	0.09
Heat	0.08
Landslide	0.06
Sum	1

In this exercise, the spatial distribution of the intensity level of heat and wind speed hazard for a five year return period was used because most of the historic climatic data does not cover a period longer than 7 years.

For the entire EM Region and the West Bank and Gaza Strip territory, the weights were then combined with the corresponding reclassified hazard distribution map using the weighted linear combination approach presented in the landslide hazard section.

The multi hazard index distribution map that resulted from this combination was then reclassified according to the five selected intensity levels as shown in Table 8.

**Table 8:** Correspondence between the multihazard index ranges and the intensity level of multihazard

**Multihazard index**	**Intensity level**
1 – 1.5	Very low
1.5 – 2.5	Low
2.5 – 3.5	Medium
3.5 – 4.5	High
4.5 – 5	Very high

**Figure 12:**
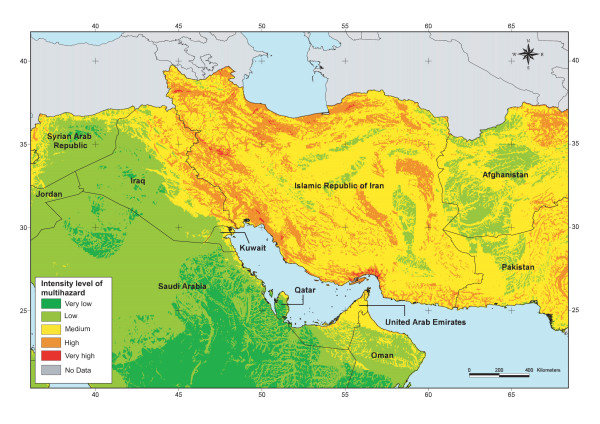
**Multihazard**. Spatial distribution of the intensity level of multihazard for part of the EM Region.

The result obtained through the application of this approach is presented for part of the EM Region in Figure 12.

### Population exposure to natural hazard

One potential application of the hazard distribution maps is the calculation of the population exposed to each of the hazard specific level of intensity.

This can be achieved through the combination of the hazard distribution layers with a population distribution grid covering the area of interest. Among the publicly available population distribution grid we can mention the 1 km resolution Landscan database [20] and the 2.5 km resolution Gridded Population of the World (GPW) data set [72].

For the present analysis, a modified version of the 2005 edition of the Landscan data set [20] was used. The modification that was done on the original grid concerns the adjustment of the total country population to make it fit the 2005 figures provide by the UN Population Division [72] and reported in Table 9.

**Table 9:** Population exposure at the regional level

**Countries**	**Total population**	**Population exposed**	**% of the total population**
**Afghanistan**	29,863,005	11,377,533	38
**Bahrain**	726,617	0	0
**Djibouti**	793,078	0	0
**Egypt**	74,032,884	1,218,398	2
**Islamic Republic of Iran**	69,515,206	40,181,472	58
**Iraq**	28,807,190	5,646,311	20
**Jordan**	5,702,776	106,953	2
**Kuwait**	2,686,873	44,791	2
**Lebanon**	3,576,818	955,908	27
**Libyan Arab Jamahiriya**	5,853,452	969,438	17
**Morocco**	31,478,460	1,207,577	4
**Oman**	2,566,981	339,985	13
**Pakistan**	157,935,075	55,730,556	35
**Qatar**	812,842	84	0
**Saudi Arabia**	24,573,100	1,154	0
**Somalia**	8,227,826	0	0
**Sudan**	36,232,945	0	0
**Syrian Arab Republic**	19,043,382	1,042,960	5
**Tunisia**	10,102,467	2,114,772	21
**United Arab Emirates**	4,495,823	302,013	7
**West Bank and Gaza Strip**	3,702,000	214,396	6
**Yemen**	20,974,655	0	0

Country-level total and percentage of population exposed to a high or very high intensity level of multihazard in the EM Region and the West Bank and Gaza Strip territory.

Using the GIS software and for each hazard, the population exposed to the different levels of intensity has been extracted for each country and is presented in the first volume of the Atlas. As an example, Table 9 summaries the distribution of the population exposed to a high or very high intensity level of multihazard in the EM Region and the West Bank and Gaza Strip territory.

When looking at this table it is important to remember that the intensity levels have been standardized for all the EM Region and the West Bank and Gaza Strip territory and that only 5 hazards are taken into account here. This table therefore only gives an indication of the relative level of exposure that exists between these countries but does not mean, for example, that the populations in Somalia or Sudan are not subject to any hazards.

Precautions should therefore being taken when using these results. We can nevertheless say that, from a regional perspective, the Eastern part of the EM Region (Afghanistan, Islamic Republic of Iran and Pakistan) and a set of countries (Iraq, Lebanon and Tunisia) are presenting the highest number and proportion of population exposed to a high or very high level of multihazard compare to the other countries.

As an other example, Table 10 present the distribution of the population of the Islamic Republic of Iran according to the different intensity levels of multihazard

**Table 10:** Population exposure at the country level

**Intensity level**	**Population exposed**	**Percentage of the total population**
Very low	0	0
Low	349,324	< 1
Medium	28,591,734	41
High	39,367,076	57
Very high	814,396	1
No data	392,676	< 1

Distribution of the population of the Islamic Republic of Iran according to the different intensity levels of multihazard

**Figure 13:**
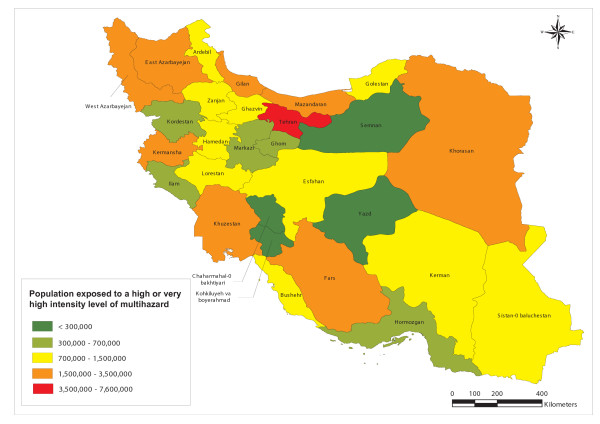
**Population exposure**. Population exposed to a high or very high level of multihazard at the provincial level in the Islamic Republic of Iran.

This approach can also be used at the sub national level. As an example, Figure 13 shows the distribution of the total population exposed to a high or very high level of multihazard at the provincial level in the Islamic Republic of Iran. In addition, Table 11 identifies the 10 provinces with the highest number of inhabitants exposed to a high or very high intensity level of multihazard in this country.

**Table 11:** Population exposure at the sub national level

**Province**	**Total population**	**Population exposed**	**Percentage of the total population (province level)**	**Percentage of the total population (country level)**
Tehran	12,030,891	7,547,300	63	19
Khuzestan	4,281,555	3,311,495	77	8
East Azarbayejan	3,837,415	3,051,983	80	8
Mazandaran	3,023,111	2,661,996	88	7
Gilan	2,574,801	2,284,661	89	6
Khorasan	7,026,625	2,228,333	32	6
Fars	4,439,231	2,091,992	47	5
West Azarbayejan	2,899,891	1,824,729	63	5
Kermansha	2,064,701	1,553,533	75	4
Golestan	1,652,543	1,442,763	87	4

Total population, population exposed and percentage of the total population (province and country level) for the 10 province with the highest number of inhabitants exposed to a high or very high level of multihazard

It is interesting to observe that in the case reported in Figure 13 the administrative unit with the highest number of inhabitants exposed to potential high or very high intensity level of multihazard is the capital city.

Even if this type of information does not account for population vulnerability (and therefore does not address risk) it is still of use to decision makers as a first proxy to determine where to target natural hazard mitigation and preparedness activities.

## Discussion and Conclusion

This paper proposes a new set of models which, using available global data sets, produce maps showing the spatial distribution of the relative intensity levels of five natural hazards (floods, heat indices, wind speed, landslides and seismic hazard) over the EM Region and the West Bank and Gaza Strip territory.

These models complement those developed by initiatives such as the Natural Disaster Hotspots (NDH) project [1] and the work done by UNDP in partnership with UNEP-GRID [2,3] by bringing the scale of analysis from global to regional and even national levels. This change of scale is achieved through the use of the following elements:

• varying return periods for climatic parameters in order to capture potential hazard events over longer period of time

• a one kilometer resolution, which has very seldom been used before in this type of project, to allow for national analysis

• use of a larger number of parameters for spatializing the distribution of each hazard in order to provide a higher level of confidence on the results

When these models are layered with population distribution data and subnational administrative boundaries, the resulting analysis can provide actionable information to decision makers working on emergency preparedness and mitigation by helping to identify and prioritize areas with high population exposure rates to high probability of hazard occurrence or intensity. From a technical perspective these models provide GIS practitioners and ultimately disaster managers with a way to refine their analytical capabilities while at the same time allowing users to create more specific, meaningful local analyses by using their own data.

Some limitations need to be addresses before making decisions on the basis of the maps resulting from the application of the proposed models. The first and main issue relates to the quality and completeness of the data used in the context of this work. More specifically:

• There are gaps and imprecision in both the river and road network data available at the time of this work.

• Some areas (i.e. small islands) are not covered by the available global data sets and the use of data from different sources resulted in shifts between some of the mapping layers. This had for consequences that the distribution of the hazard intensity level could not be determined in some populated areas.

• The lack of historical meteorological data for several countries (Afghanistan, Iraq and Somalia) reduces the confidence in the analysis for those areas.

• Important differences in nomenclature are observed between countries with regard to the geological layer and this can affect the results of the analysis.

• The resolution of the output hazard distribution maps has been set to 1 km. Even if there is no absolute rules it is important to keep in mind that the validity of this choice is function of the resolution of the input data. For heat and wind speed the resolution of the selected input variables support this decision while, in the case of landslide and flood as well as for the seismic hazard, the use of data sets presenting scales varying between 1:2'000'000 and 1:12'000'000 confirms the need to consider the results with cautions if to be used locally.

In addition to that it is important to mention that the one kilometer resolution may mask some micro-variations (such as crucial elevation changes in the topography in an area that could be trigger some local landslide or floods) that have direct impact on the occurrence or intensity of the hazards.

Access to more complete, more accurate data from other sources would help solve these issues and could increase the quality of the results obtained through the application of the proposed models.

The second issue regards the application of the models themselves, mainly:

• The fact that we divided the study area into 4 zones when modelling the spatial distribution of the different climatic variables induced some discontinuities at the border between these zones. This is, for example visible when looking at the wind speed hazard distribution maps for a five or ten year return period (Figure 7b and 7c).

• In its current form the generation of the flow accumulation layers requires not to have any holes in the Digital Elevation Model (DEM). This operation nevertheless generates flat surfaces which results in artefacts that appear in the resulting flood hazard distribution grid.

• Because of the lack of information based on outcome, the method used for landslide actually measured susceptibility and not hazard. This observation can be extended to the other hazard as well because in general, the information regarding past events is very limited.

• For heat, the approach used look at the annual daily maximum heat index while the most important impact on population occurs when a certain threshold is passed during 3 consecutive days (heat wave). The maps presented here therefore tends to overestimate heat hazard.

• Ideally, the results obtained through the application of these models should be validated, either through statistical analysis or ground truthing in the field. This has so far not been done.

• Finally, more regional and national studies should be conducted especially where better data and/or local expertise are available.

Based upon these limitations, the results obtained from these models should be considered as a way to identify the areas where greater focus on emergency preparedness and mitigation should be placed and, at this point in time, not as quantitatively descriptive of the hazards and the associated processes.

The next step of this exercise will be to further develop the health component of the Atlas and produce additional volumes by mapping the distribution of population and structural vulnerabilities, in order to target disaster preparedness programmatic planning where it is most needed. Subsequent volumes of the Atlas will also address issues such as accessibility to health services. This component will be quantified from a geographic perspective using GIS based tools such as AccessMod ^© ^[74]. In order to illustrate results at a district level the additional volumes will include complete and updated administrative boundaries data sets for the EM Region developed through the Second Administrative Level Boundaries (SALB) data set project [13].

The ability to reduce the risks/impact of natural hazards remains a challenge to many institutions and organizations and it is anticipated that the results presented here, in conjunction with disaster preparedness programs and activities, will contribute to addressing this need. In this regard, WHO is looking forward, through this initiative, to working with already established networks of collaborators. Among those networks we can mention the recently launched Global Risk Identification Program (GRIP) [75] which facilitates knowledge and data sharing between numerous stakeholders in an effort to strengthen existing models and data sets and to develop an integrated approach to emergency management at all levels. Other UN agencies such as the World Food Program (WFP) also have similar tools targeting food security in high risk countries. Strong efforts will be made to ensure the coordination and sharing of such vital data in order to develop a comprehensive vulnerability profile for the countries part of the EM Region and the West Bank and Gaza Strip territory.

## Authors' contributions

ZEM reviewed the preliminary hazard models, developed the final ones and applied them to the EM Region and the West Bank and Gaza Strip territory, and contributed to the drafting and revision of the manuscript.

SE coordinated the design and management of the study, reviewed the preliminary hazard models, contributed to the development of the final models and to the writing and revision of the manuscript.

JB developed the preliminary hazard models, applied them to selected areas of the EM Region and the West Bank and Gaza Strip territory, and contributed to the revision of the manuscript.

EAG contributed and provided guidance in the development of the preliminary hazard models and contributed to the drafting and revision of the manuscript.

AM conceived of the study, coordinated the development of the preliminary hazard models and contributed to the review and revision of the manuscript.

## Disclaimer

The designations employed and the presentation of the material in this publication do not imply the expression of any opinion whatsoever on the part of the World Health Organization concerning the legal status of any country, territory, city or area or of its authorities, or concerning the delimitation of its frontiers or boundaries. Dotted lines on maps represent approximate border lines for which there may not yet be full agreement.

All reasonable precautions have been taken by WHO to verify the information contained in this publication, However, the Atlas is being distributed without warranty of any kind, either express or implied regarding its content. The responsibility for its interpretation and use lies with the user. In no event shall the of hazard
